# Mesenchymal stem cells: a potential therapeutic strategy for IgA nephropathy

**DOI:** 10.3389/fphar.2026.1885100

**Published:** 2026-09-22

**Authors:** Jia Lv, Huan Yang, Na Tian, Shi Cheng, Xin Lu, Xuehong Lu

**Affiliations:** 1 Department of Nephrology, The Second Hospital of Jilin University, Changchun, China; 2 Jilin Tuohua Biotechnology Co., Ltd., Siping, China; 3 Department of Nephrology, People’s Hospital of Jilin City, Jilin, China

**Keywords:** IgA nephropathy, immunosuppressive therapy, mesenchymal stem cells, multi-organ mechanism, supportive care

## Abstract

IgA nephropathy (IgAN) is the predominant primary glomerulonephritis worldwide, characterized by the deposition of IgA-containing immune complexes in the glomerular mesangium. Despite advances in supportive care, there is a lack of IgAN-specific therapies capable of halting IgAN progression. Mesenchymal stem cells (MSCs) have emerged as a promising therapeutic target, owing to their immunomodulatory, anti-inflammatory, and tissue repair properties. Accumulating evidence suggests that MSCs hold considerable therapeutic potential for the treatment of IgAN. This review summarizes the potential mechanisms underlying the therapeutic effects of MSCs in IgAN, with a particular focus on their roles in the gut, liver, and kidney, and discusses current challenges and future directions for MSCs-based therapies.

## 1 Introduction

IgA nephropathy (IgAN) is a predominant primary glomerular disease worldwide and a major cause of chronic kidney disease (CKD) and end-stage renal disease (ESRD) (Ghaddar et al., 2024). Epidemiological studies have indicated that approximately 30%–50% of IgAN cases progress to ESRD within 10–20 years after diagnosis, accompanied by a significantly reduced life expectancy compared to that of the general population (Jarrick et al., 2019). The currently accepted pathogenesis of IgAN is the “multi-hit hypothesis.” According to this model, dysregulated mucosal immunity promotes the activation of mucosal B cells through interactions with T follicular helper (Tfh) cells and the cytokines APRIL and BAFF, resulting in the production of galactose-deficient IgA1 (Gd-IgA1). Gd-IgA1 subsequently enters the circulation and binds anti-Gd-IgA1 autoantibodies to form pathogenic immune complexes. These complexes deposit in the glomerular mesangium, triggering complement activation, local inflammation, and mesangial cell proliferation. Activated mesangial cells further release inflammatory mediators that promote podocyte injury and progressive renal fibrosis, ultimately leading to glomerular injury and renal dysfunction (van Egmond et al., 2000; Tomana et al., 1997; Gao et al., 2024). In recent years, the therapeutic landscape of IgAN has evolved substantially with the emergence of several disease-modifying therapies, including sodium–glucose cotransporter 2 (SGLT2) inhibitors (Caravaca-Fontán et al., 2024), targeted-release budesonide (Barratt et al., 2023), complement inhibitors (Schubart et al., 2019), and B-cell-targeted therapies (Lv et al., 2026). These agents have demonstrated promising efficacy in reducing proteinuria and slowing renal function decline. However, their mechanisms of action, therapeutic benefits, and safety profiles differ substantially. While some immunosuppressive therapies may be associated with clinically important adverse effects, newer targeted therapies exhibit distinct and treatment-specific safety profiles (Lv et al., 2022; Lafayette et al., 2023). Moreover, most currently available therapies primarily target specific components of the pathogenic cascade. In this context, MSCs-based therapy has attracted increasing interest because of its potential to exert pleiotropic immunomodulatory and tissue-protective effects across multiple levels of the gut–liver–kidney axis (He et al., 2026; Liu et al., 2026). Therefore, this review focuses specifically on the mechanistic rationale and current evidence supporting MSCs therapy in IgAN.

MSCs are multipotent stromal cells of mesodermal origin that can be isolated from various tissues, including bone marrow, adipose tissue, and the umbilical cord (Mosna et al., 2010; Zuk et al., 2002; Chamberlain et al., 2007; Yu et al., 2025). MSCs exhibit low immunogenicity and potent immunomodulatory properties, enabling their use in allogeneic transplantation without significant immune rejection (Ankrum et al., 2014; Li et al., 2012). In addition, MSCs possess homing capabilities, allowing them to migrate to sites of inflammation or tissue injury (Karp and Teol, 2009), where they can promote tissue repair and immune regulation through paracrine signaling mediated by various cytokines and growth factors (Chen et al., 2008; Sémont et al., 2013; Wu et al., 2007; Ma et al., 2014). Due to these characteristics, MSCs have attracted much attention in recent years for the treatment of various inflammatory and immune-related diseases.

Early studies suggested that IgAN may be associated with stem cell dysfunction. Imasawa et al. demonstrated that transplantation of bone marrow from normal C57BL/6J mice into HIGA mice reduced serum IgA levels and attenuated mesangial IgA and C3 deposition, compared with HIGA mice receiving bone marrow from syngeneic HIGA donors (Imasawa et al., 1999). This study suggests that hematopoietic stem cell abnormalities may be involved in the pathogenesis of IgAN. More recent animal studies and preliminary clinical investigations have shown that MSCs therapy can attenuate mesangial proliferation, reduce renal fibrosis, and significantly decrease proteinuria in IgAN (Zhang et al., 2015; Tanaka et al., 2022; Hyun et al., 2012). Furthermore, case reports have indicated that intravenous infusion of human umbilical cord-derived MSCs can improve renal function and clinical symptoms in patients with IgAN (Riordan et al., 2022).

Collectively, these findings suggest that MSCs have the potential to treat IgAN. Building on these observations, this review examines the potential effects of MSCs across the interconnected gut–liver–kidney axis within the pathogenic cascade of IgAN (Figure 1). In contrast to previous reviews, we further distinguish direct evidence in IgAN from indirect evidence and hypothesis-level mechanisms to highlight current evidence gaps and future research priorities.

**FIGURE 1:**
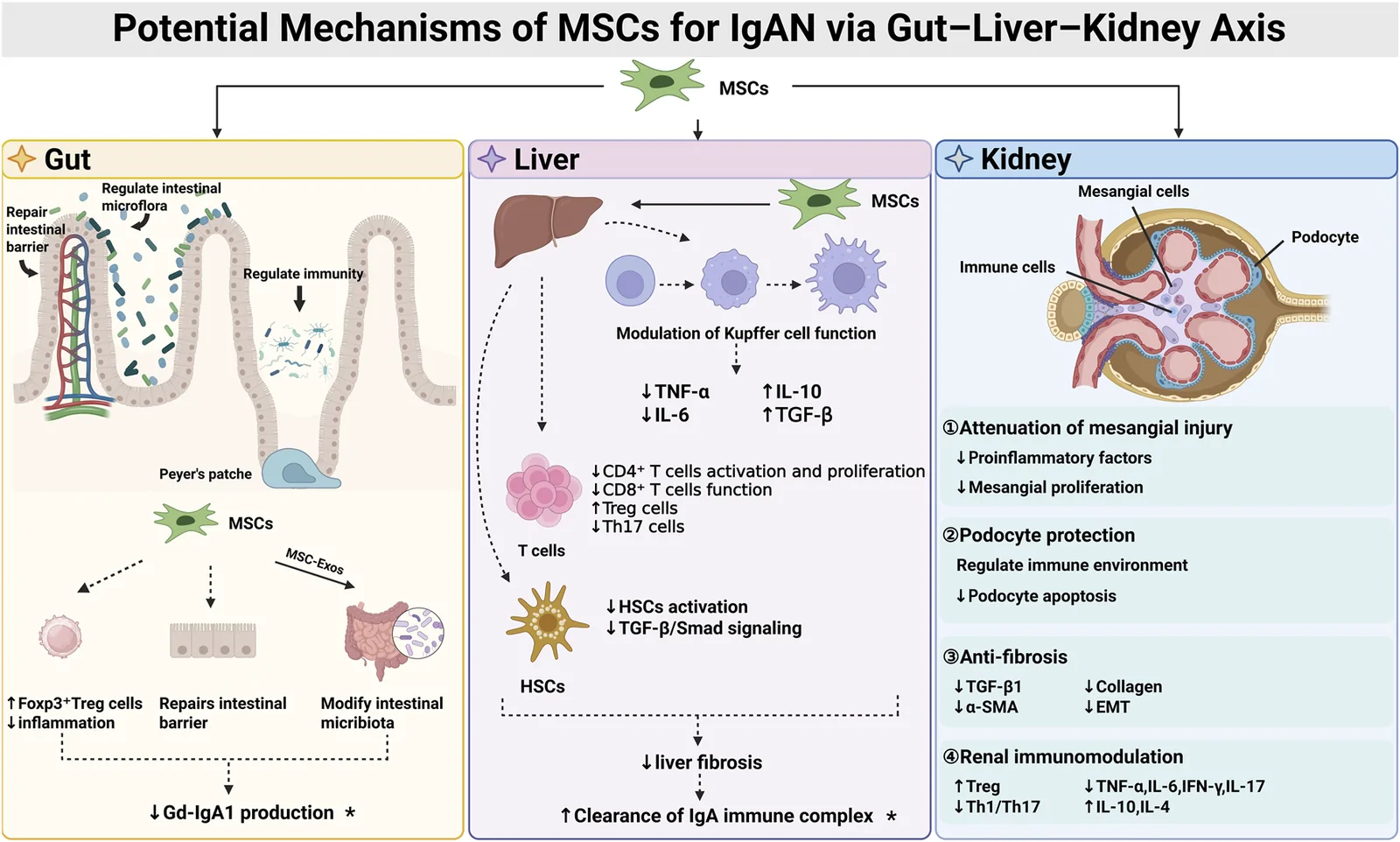
Potential multi-organ mechanisms of MSCs in IgAN across the Gut-Liver-Kidney axis. Solid lines indicate direct evidence in IgAN; dashed lines indicate hypothetical mechanisms in IgAN; * indicates proposed effects not directly validated in IgAN.

## 2 Potential mechanisms of MSCs in the gut in IgAN

Emerging evidence highlights the critical role of the intestinal mucosal immune system in the pathogenesis of IgAN. Impairment of the intestinal barrier and increased intestinal permeability are considered key factors that trigger aberrant mucosal immune responses. Clinical studies have shown that several inflammatory intestinal diseases, such as celiac disease, ulcerative colitis, and Crohn’s disease, frequently coexist with IgAN (Collin et al., 2002; Onime et al., 2006; Takemura et al., 2002), suggesting a shared pathogenic mechanism involving mucosal immune dysregulation. Moreover, the fact that experimental models of IgAN can be induced by oral mucosal immunization further supports this concept (Meng T. et al., 2014; Yang L. et al., 2018; He et al., 2015).

When the intestinal barrier is compromised, luminal antigens and microbial products can more readily translocate across the epithelium, activating mucosa-associated lymphoid tissue (MALT) and inducing abnormal mucosal immune responses (Tang et al., 2022). In addition, endotoxins and microbial components entering the circulation may promote chronic low-grade inflammation and stimulate the overproduction of aberrant IgA. Subsequently, excess Gd-IgA1 forms immune complexes that deposit in the glomerular mesangium, contributing to disease onset and progression. These findings have led to the concept of the “gut–kidney axis” as an explanation for the role of intestinal immunity in IgAN (Monteiro and Berthelot, 2021; Yang T. et al., 2018).

Studies on MSCs therapy for inflammatory bowel disease (IBD) have provided important insights into their potential application in IgAN. MSCs can promote intestinal barrier repair through multiple mechanisms. Specifically, MSCs migrate to inflamed intestinal tissues and secrete growth factors and cytokines that enhance epithelial regeneration, thereby restoring barrier integrity (Lian et al., 2011; Liang et al., 2023; Sun et al., 2024). Moreover, MSCs modulate the local immune microenvironment via paracrine effects, suppressing inflammation and promoting tissue repair (Guo et al., 2022; Mao et al., 2017; Jiang et al., 2024). In addition, MSCs can regulate the composition of the gut microbiota. In murine models of colitis, MSCs treatment significantly improves microbial diversity and restores microbiota composition to a near-normal state, as well as reducing intestinal inflammation (Liu et al., 2022). Notably, a recent study demonstrated that human umbilical cord mesenchymal stem cell–derived exosomes (hUCMSC-Exos) altered gut microbiota composition and modulated inflammatory signaling in experimental IgAN, providing direct evidence that MSCs-derived biological products can influence gut-associated pathogenic processes in this disease (He et al., 2026). However, these findings were obtained using exosomes rather than MSCs themselves and should therefore be interpreted separately from cellular therapy studies.

Within the intestinal mucosal immune system, Peyer’s patches serve as key sites for IgA production (Huang et al., 2024). B cells in Peyer’s patches differentiate into IgA-secreting plasma cells through both T cell-dependent and independent pathways, maintaining mucosal immune homeostasis (Reboldi et al., 2016). Regulatory T cells (Tregs) play a crucial role in this process (Yan et al., 2023). MSCs can increase the proportion of Foxp3^+^ Treg cells in Peyer’s patches, thereby suppressing excessive inflammatory responses and maintaining immune tolerance (Liu et al., 2022).

IgAN and IBD share several features of mucosal dysregulation, including altered gut microbial communities, aberrant mucosal immune responses, and impaired intestinal barrier homeostasis (Yi et al., 2026; Terasaka et al., 2016; Sanchez-Russo et al., 2022; Doumas et al., 2023). Although accumulating evidence supports the beneficial effects of MSCs on intestinal barrier function and mucosal immune homeostasis in IBD, there is no direct evidence that MSCs can reduce the production of Gd-IgA1 or correct abnormal IgA glycosylation in IgAN.

## 3 Potential mechanisms of MSCs in the liver in IgAN

The liver plays a central role in IgA metabolism and clearance and is a key organ for maintaining circulating IgA homeostasis (Brown and Kloppel, 1989). Kupffer cells within hepatic sinusoids can recognize and phagocytose circulating IgA and IgA-containing immune complexes via Fcα receptors, thereby facilitating their degradation and clearance (Rifai and Mannik, 1984; Xu et al., 2016).

In patients with IgAN, hepatic immune clearance function can be impaired, leading to the persistent accumulation of IgA and its immune complexes in the circulation. Reduced clearance efficiency increases the likelihood of pathogenic IgA deposition in the glomerular mesangium, which triggers inflammatory responses (Chen et al., 2018). Furthermore, clinical studies have demonstrated that patients with chronic liver diseases frequently develop secondary IgA deposition nephropathy (Kanduri et al., 2024; Umezawa et al., 2025), further supporting the involvement of hepatic dysfunction in IgAN pathogenesis. Therefore, restoring hepatic immune clearance capacity may represent a promising therapeutic strategy for IgAN.

A growing body of evidence indicates that MSCs possess significant therapeutic potential for various liver diseases. MSCs can improve the hepatic inflammatory microenvironment and promote hepatocyte regeneration through the secretion of cytokines and growth factors (Huang et al., 2025). In addition, MSCs can suppress Kupffer cell overactivation and regulate T cell responses, thereby alleviating inflammation and restoring immune homeostasis (You et al., 2015; Tian et al., 2016). Therefore, the liver is another potentially important site for MSCs to influence disease progression: MSCs may facilitate the removal of circulating IgA and immune complexes by modulating the immune microenvironment of the liver.

Mechanistically, MSCs regulate Kupffer cell function via signaling pathways involving tumor necrosis factor-α (TNF-α) and prostaglandin E2 (PGE2), resulting in decreased production of pro-inflammatory cytokines (e.g., IL-1β and IL-6) and increased secretion of anti-inflammatory mediators (e.g., IL-10 and TGF-β) (You et al., 2015; Sitbon et al., 2024). In models of liver fibrosis and chronic liver injury, MSCs inhibit the activation of hepatic stellate cells (HSCs) and suppress the TGF-β/Smad signaling pathway, thereby reducing extracellular matrix deposition and attenuating fibrosis (Chiabotto et al., 2024). Additionally, MSCs exert immunomodulatory effects by regulating T cell responses within the hepatic microenvironment. MSCs may suppress the activation and proliferation of CD4^+^ T cells, inhibit the effector functions of CD8^+^ T cells, promote the expansion of regulatory T cells (Tregs), and suppress Th17-mediated inflammatory responses, thereby rebalancing T cell immunity (Hu and Li, 2019; Hu et al., 2020). This process contributes to reducing pro-inflammatory immune responses and promotes the establishment of immune tolerance.

Overall, given that the liver is the primary organ responsible for clearing circulating IgA and IgA-containing immune complexes, MSCs-mediated liver repair has the potential to enhance the clearance of circulating IgA and immune complexes. Meanwhile, regulation of the liver immune microenvironment by MSCs may alleviate immune dysregulation driven by inflammation and may indirectly inhibit immune complex formation and deposition. However, there is no direct evidence that MSCs can promote the clearance of immune complexes in IgAN patients. Therefore, further research is needed to confirm whether MSCs can promote IgA uptake by Kupffer cells or enhance the ability to clear immune complexes from the blood.

## 4 Potential mechanisms of MSCs in the kidneys in IgAN

Once pathogenic immune complexes are deposited within the glomerulus, persistent complement activation and local inflammatory responses drive mesangial-cell activation, podocyte injury, and progressive renal fibrosis. At this downstream stage of the disease process, MSCs may primarily exert renoprotective effects by modulating renal immune responses and directly protecting intrinsic renal cells.

### 4.1 Immunomodulatory mechanisms

IgAN is a prototypical immune-mediated glomerular disease, the pathogenesis of which involves imbalances in both innate and adaptive immunity, including T-cell subset dysregulation, aberrant macrophage polarization, and excessive release of inflammatory cytokines (Cheung et al., 2025). Owing to their potent immunomodulatory capacity, MSCs play a central role in reshaping the renal immune microenvironment. First, MSCs are capable of restoring T-cell immune homeostasis. Accumulating evidence indicates that MSCs inhibit the activation and proliferation of CD4^+^T cells, suppress pro-inflammatory responses mediated by Th1 and Th17 cells, and promote the expansion of regulatory T cells (Tregs), thereby re-establishing immune tolerance (Li et al., 2025; Müller et al., 2021). Correction of the Th17/Treg imbalance is considered a critical step in the alleviation of inflammatory responses in IgAN (Yi et al., 2025). IL-17 secretion by Th17 cells is significantly elevated in IgAN (Zhou et al., 2025). Inhibition of IL-17 expression ameliorated mesangial lesions (Watanabe et al., 2022) have been reported to reduce IL-17 secretion (Montenegro et al., 2024). Therefore, modulation of IL-17 signaling may represent a potential mechanism through which MSCs alleviate pathological changes in IgAN.

Second, MSCs exert significant regulatory effects on the innate immune system. They can induce macrophage polarization toward the anti-inflammatory M2 phenotype, leading to reduced secretion of pro-inflammatory cytokines, such as TNF-α and IL-6, while enhancing the expression of anti-inflammatory cytokines, such as IL-10 (Zhu et al., 2025; Lotfinejad et al., 2021). This phenotypic shift contributes to the attenuation of local glomerular inflammation and limits the amplification of inflammatory cascades.

In summary, by restoring T-cell homeostasis and promoting anti-inflammatory macrophage polarization, MSCs may establish an anti-inflammatory immune microenvironment and ultimately suppress immune-mediated renal injury in IgAN.

### 4.2 Kidney-specific cellular and tissue protection mechanisms

In addition to immune regulation, MSCs can also act directly on kidney cells, including mesangial cells, podocytes, and interstitial cells, thereby alleviating structural damage and delaying disease progression.

Mesangial cell proliferation and excessive extracellular matrix deposition are key pathological features of IgAN (Zhang et al., 2023). Following immune complex deposition, mesangial cells become activated and secrete various inflammatory mediators and growth factors, such as IL-6, platelet-derived growth factor (PDGF), and TGF-β, promoting cell proliferation and matrix expansion (Zhang et al., 2023; Liang et al., 2015). MSCs can directly attenuate mesangial cell injury in IgAN. In HIGA mice (a spontaneous IgAN animal model), adipose-derived MSCs significantly improved renal function, reduced mesangial hypercellularity, and alleviated glomerular pathological injury (Hyun et al., 2012). These protective effects were accompanied by decreased expression of pro-inflammatory cytokines and suppression of mesangial cell activation, suggesting that MSCs may directly interfere with the downstream pathogenic consequences of mesangial immune-complex deposition. Recent studies further suggest that the renoprotective effects of MSCs in IgAN may involve the regulation of inflammatory signaling pathways, restoration of cellular homeostasis, and inhibition of pathological mesangial-cell proliferation (Liu et al., 2026). By suppressing the production of inflammatory mediators and limiting excessive matrix deposition, MSCs may interrupt the vicious cycle linking mesangial activation, chronic inflammation, and progressive renal injury.

Podocytes are essential for maintaining the integrity of the glomerular filtration barrier, and their injury or loss is closely associated with proteinuria in IgAN (Xu et al., 2010). Activated mesangial cells can release inflammatory mediators that induce podocyte apoptosis and cytoskeletal damage (Zhu et al., 2013). MSCs exert protective effects on podocytes primarily through paracrine mechanisms. MSCs-conditioned medium has been shown to significantly reduce podocyte apoptosis and restore the expression of key slit diaphragm proteins, such as nephrin and synaptopodin (Li et al., 2013). Furthermore, MSCs-secreted growth factors—including epidermal growth factor (EGF), hepatocyte growth factor (HGF), and insulin-like growth factor-1 (IGF-1)—contribute to maintaining podocyte structure and function (Li et al., 2013). MSCs also provide indirect protection by modulating the renal immune microenvironment. Specifically, MSCs-induced macrophage polarization toward the M2 phenotype reduces inflammation-induced podocyte injury (Zhu et al., 2025). Some studies have also demonstrated that MSCs can enhance podocyte autophagy via activation of the SIRT1/FOXO1 signaling pathway, improving resistance to cellular stress (Liu et al., 2024).

Renal fibrosis is the final common pathway of CKD progression and a major determinant of renal function decline in IgAN (Fu et al., 2024). Persistent inflammation and glomerular injury drive fibroblast activation and excessive extracellular matrix deposition (Meng X M. et al., 2014). MSCs exhibit potent anti-fibrotic effects in various kidney disease models and can reduce proteinuria and improve renal histological damage (Lv et al., 2024; Imberti et al., 2020). Furthermore, they can significantly reduce the expression of fibrosis-related markers, including α-smooth muscle actin (α-SMA), collagen, and TGF-β1, thereby alleviating renal fibrosis and improving histopathological injury (Lv et al., 2024). In addition, MSCs-secreted factors, such as HGF and vascular endothelial growth factor (VEGF), promote tissue repair and angiogenesis, further inhibiting fibrotic progression (Imafuku et al., 2019).

Although evidence supports the protective effects of MSCs in IgAN, the mechanisms underlying these effects remain largely unresolved. Current studies demonstrate that MSCs suppress renal inflammation, inhibit mesangial-cell activation, protect podocytes, and attenuate renal fibrosis (Liu et al., 2026). Consistent with these observations, a recent study showed that hUCMSC-Exos improved renal injury markers and attenuated renal pathological changes in experimental IgAN, suggesting that MSCs-derived biological products may also exert renoprotective effects (He et al., 2026). Nevertheless, because these findings were obtained using exosomes rather than MSCs themselves, they should be interpreted separately from evidence derived from cellular therapy. However, direct evidence regarding the effects of MSCs on Gd-IgA1 production, anti-Gd-IgA1 autoantibody generation, pathogenic immune-complex formation, and complement activation is still lacking. Future studies integrating disease-specific biomarkers with mechanistic analyses will be essential to clarify the precise therapeutic targets of MSCs in IgAN.

## 5 Integrated mechanistic model of MSCs-mediated gut–liver–kidney regulation in IgAN

Although current evidence regarding MSCs therapy in IgAN is often discussed within separate intestinal, hepatic, and renal contexts (Table 1), these mechanisms may be interconnected through a broader gut–liver–kidney regulatory network. Following MSCs administration, restoration of intestinal immune homeostasis and barrier integrity may reduce the translocation of microbial products and inflammatory mediators into the portal circulation. Consequently, the inflammatory burden reaching the liver may be diminished, contributing to a more balanced hepatic immune microenvironment. Given the central role of the liver in IgA metabolism and immune-complex handling (Perše and Večerić-Haler, 2019), improved hepatic homeostasis may theoretically favor the physiological processing of circulating IgA and IgA-containing immune complexes. Although direct evidence demonstrating enhanced hepatic clearance of pathogenic IgA complexes following MSCs therapy is currently lacking, this mechanism represents a plausible link between intestinal immune regulation and downstream renal protection. A reduced burden of circulating pathogenic immune complexes could subsequently attenuate mesangial activation, glomerular inflammation, and progressive kidney injury (Knoppova et al., 2021). In parallel, MSCs may exert direct renoprotective effects by suppressing mesangial-cell proliferation, protecting podocytes, and limiting renal fibrosis. Therefore, rather than acting on isolated organs, MSCs therapy may function through coordinated regulation of interconnected intestinal, hepatic, and renal pathways, forming a putative gut–liver–kidney regulatory network in IgAN.

**TABLE 1:** Evidence level supporting the proposed mechanisms of MSCs-based therapies across the pathogenic cascade of IgAN.

Pathogenic process	Proposed mechanism of MSCs therapy	Study type	Evidence presented	Evidence level
Intestinal immune homeostasis	Regulation of intestinal immune responses and inflammatory microenvironment	*In vivo* and clinical	Supported primarily by IBD models (Guo et al., 2022; Mao et al., 2017; Jiang et al., 2024)	Indirect evidence
Intestinal barrier integrity	Restoration of epithelial barrier function	*In vivo*, *in vitro*, and clinical	Supported primarily by IBD models (Lian et al., 2011; Liang et al., 2023; Sun et al., 2024)	Indirect evidence
Gut microbiota dysbiosis	Remodeling of gut microbiota composition and alterations in inflammatory signaling	*In vivo* and *in vitro*	Supported primarily by IBD models (Liu et al., 2022) and IgAN models treated with hUCMSC-Exos (He et al., 2026)	Indirect evidence (MSCs); Direct IgAN evidence (MSC-Exos)
Liver immune regulation and IgA clearance	Modulation of liver immune microenvironment and potential enhancement of IgA immune-complex clearance	*In vivo* and *in vitro*	Liver clearance of IgA has been proven (Chen et al., 2018; Kanduri et al., 2024; Umezawa et al., 2025), but no direct evidence demonstrates that MSCs enhance this process in IgAN	Hypothesis
Mesangial cell activation and proliferation	Suppression of mesangial-cell proliferation and inflammatory responses	*In vivo*, *in vitro*, and clinical	*In vivo* studies demonstrated decreased mesangial proliferation, while *in vitro* studies showed reduced inflammatory responses in mesangial cells (Hyun et al., 2012). Preliminary clinical evidence has also reported reduced mesangial proliferation in IgAN (Cho et al., 2025).	Direct IgAN evidence
Podocyte injury	Protection against podocyte injury	*In vivo* and *in vitro*	Supported mainly by diabetic nephropathy and other chronic kidney disease models (Li et al., 2013; Liu et al., 2024)	Indirect evidence
Renal fibrosis	Attenuation of renal fibrosis	*In vivo*	Supported by studies in chronic kidney disease and experimental renal fibrosis (Lv et al., 2024; Imberti et al., 2020)	Indirect evidence
Renal injury and inflammation	Reduction of renal injury and inflammatory signaling	*In vivo*	IgAN models treated with hUCMSC-Exos showed improved renal injury markers and attenuated renal pathological changes (He et al., 2026)	Direct IgAN evidence (MSC-Exos)

Evidence levels: Direct IgAN, evidence, findings directly validated in IgAN; indirect evidence, findings supported by non-IgAN, models or related biological contexts; Hypothesis, biologically plausible mechanisms without direct or indirect experimental evidence.

## 6 Clinical evidence and translational potential of MSCs-Based therapy in IgAN

### 6.1 Early clinical studies and safety profiles

To date, clinical evidence on MSCs therapy in IgAN remains extremely limited but promising. A phase I, open-label, dose-escalation study evaluating the safety and tolerability of MSCs-based therapy in patients with IgAN demonstrated that MSCs infusion was generally well tolerated, with no serious adverse events directly attributable to the treatment (Tanaka et al., 2022; Tanaka et al., 2024). Importantly, no evidence of acute immune rejection or tumorigenicity was observed during the follow-up period, supporting the favorable safety profile of MSCs therapy in clinical settings (Ankrum et al., 2014; Li et al., 2012; Tanaka et al., 2022). However, the study involved only a small number of participants and was not designed to evaluate long-term efficacy or hard renal outcomes. In addition, case reports have described improvements in clinical parameters following MSCs therapy in patients with IgAN, including reductions in serum creatinine levels, improvements in hematocrit and hemoglobin levels, and remission of clinical symptoms (Riordan et al., 2022). Nevertheless, these observations were derived from uncontrolled studies with very limited sample sizes and therefore should be regarded as preliminary proof-of-concept findings rather than definitive evidence of therapeutic efficacy (Table 2).

**TABLE 2:** Clinical trials on mesenchymal stem cell therapy in IgAN.

Study/name of the trial	Study design	Number of patients	Cell type	Delivery method	Cell dose	Follow-up period	Outcomes	Side effects in cell-treated patients	Reference number
ADR-001 (2024)	Nonrandomized, uncontrolled study	9	Adipose-derived MSCs	Infusion intravenously	Cohort 1: a single dose of 1 × 10^8^ cells; Cohort 2: two doses of 1 × 10^8^ cells, 2-week interval	Screening period: approximately 4 weeks; observation period: 52 weeks	Kidney injury markers and urine protein levels↓	No major complications reported	Tanaka et al. (2024)
Riordan NH et al. (2022)	Case report	1	Umbilical cord-derived mesenchymal stem cells	Vein infusion	A total of 120 million	6 months	Creatinine levels↓ Hematocrit and hemoglobin levels improved	No major complications reported	Riordan et al. (2022)

Beyond IgAN, extensive clinical experience with MSCs in other immune-mediated and inflammatory diseases—such as graft-versus-host disease, systemic lupus erythematosus, and inflammatory bowel disease—further supports their safety and immunomodulatory efficacy (Müller et al., 2021; Yi et al., 2025; Lotfinejad et al., 2021; Zou et al., 2016; Bianchi et al., 2014; Le Blanc and Ringdén, 2007; Wang et al., 2016; Galipeau and Sensébé, 2018). While these findings provide a rationale for further investigation of MSCs-based therapies in IgAN, the long-term efficacy, optimal treatment protocols, and clinical positioning of MSCs relative to emerging disease-modifying therapies remain to be established through adequately powered randomized controlled trials.

### 6.2 Challenges in clinical translation

An important consideration for the clinical translation of MSCs therapy is the biodistribution of intravenously administered cells. Following systemic infusion, the majority of MSCs are initially retained within the pulmonary microvasculature due to the pulmonary first-pass effect, while only a small proportion subsequently reaches peripheral organs such as the liver, kidney, or intestine (Gholamrezanezhad et al., 2011; Sanchez-Diaz et al., 2021). Nevertheless, accumulating evidence suggests that the therapeutic efficacy of MSCs depends not only on direct tissue engraftment but also on their systemic immunomodulatory and paracrine activities (Alvites et al., 2022; Fan et al., 2020). Soluble mediators produced by MSCs, together with secondary modulation of circulating immune cells, may exert biological effects at distant sites despite limited long-term tissue retention. However, the biodistribution, persistence, and organ-specific mechanisms of MSCs in IgAN remain insufficiently characterized and require further investigation.

Furthermore, heterogeneity among MSCs derived from different tissue sources, donors, and culture conditions may result in variability in therapeutic efficacy (Chiabotto et al., 2024; Yi et al., 2025; Phinney and Pittenger, 2017). Thus, standardization of MSCs preparation, characterization, and quality control is essential (Dominici et al., 2006). Another challenge lies in optimizing treatment protocols, including the selection of cell sources, dosing regimen, route of administration, and timing of intervention (Squillaro et al., 2016). Currently, there is no consensus on the optimal therapeutic strategy. Therefore, further studies are needed to define standardized protocols.

Moreover, long-term safety and efficacy data remain lacking. Although short-term studies suggest that MSCs therapy is safe, potential risks such as immunosuppression-related infections, ectopic tissue formation, and tumor-promoting effects require careful evaluation in large-scale, long-term clinical trials (Ankrum et al., 2014; Li et al., 2012; Tögel and Westenfelder, 2010). Finally, the high cost and complexity of MSCs production may limit their widespread clinical application (Heathman et al., 2015). The development of scalable and cost-effective manufacturing processes, as well as the advancement of EV-based therapies, may help address these challenges.

### 6.3 Future directions

Future research should focus on conducting large-scale, randomized controlled trials to establish the efficacy and safety of MSCs-based therapies for IgAN (Tanaka et al., 2022; Cheung et al., 2025; Rovin et al., 2021). In addition, advances in single-cell sequencing, multi-omics technologies, and bioengineering approaches may provide deeper insights into the mechanisms of MSC action and facilitate the development of more precise and personalized therapeutic strategies (Stuart and Satija, 2019; Hasin et al., 2017; Xiang et al., 2022).

The combination of MSCs therapy with existing treatments, such as immunosuppressive agents or gut-targeted therapies, also represents a promising avenue for enhancing therapeutic efficacy (Lafayette et al., 2023; Fellström et al., 2017). Furthermore, the development of engineered MSCs or EVs with enhanced targeting and immunomodulatory capabilities may further improve clinical outcomes (Vader et al., 2016; Samir et al., 2013; Lener et al., 2015; Wiklander et al., 2019).

## 7 Conclusion

MSCs represent a promising therapeutic strategy for IgAN because of their potential to exert multi-target immunomodulatory and tissue-protective effects. However, the strength of evidence supporting their proposed mechanisms varies considerably across different stages of IgAN pathogenesis. Current evidence more directly supports the renoprotective effects of MSCs, whereas many proposed intestinal and hepatic mechanisms are derived from indirect evidence or remain hypothetical. In particular, whether MSCs directly regulate Gd-IgA1 production, anti-Gd-IgA1 autoantibody generation, pathogenic immune-complex formation, complement activation, or hepatic clearance of IgA-containing immune complexes remains unresolved. Future studies should therefore focus on validating these mechanisms using disease-specific models and biomarkers. In parallel, standardized MSCs products and treatment protocols, together with adequately powered clinical trials, will be required to establish their long-term efficacy, safety, and clinical positioning in IgAN.
